# The evaluation of perfusion index as a predictor of vasopressor requirement in patient with sever sepsis and septic shock

**DOI:** 10.1186/2197-425X-3-S1-A230

**Published:** 2015-10-01

**Authors:** I Rasmy, N Nabil, H Mohamed, S Abdel Raouf, A Hasanin, A Eladawy, M Ahmed, A Mukhtar

**Affiliations:** Cairo University, Cairo, Egypt

Despite various campaigns and the presence of strong evidence for management, septic shock remains the leading cause of death worldwide [1]. A strong association has been found between early initiations of vasopressor therapy and reduced mortality in these patients [2]. These findings raise an important question: Is there any tool that can predict the requirement of vasopressor therapy in patients with severe sepsis.

## Objectives

A prospective observational study was conducted to examine whether perfusion index (PI) could predict vasopressor use during early resuscitation of patients with severe sepsis.

## Methods

All consecutive patients who were clinically suspected of having severe sepsis defined by the criteria of the American College of Chest Physicians/ Society of Critical Care Medicine Consensus Conference; were included. Upon admission to intensive care unit (ICU), hemodynamic, central and peripheral perfusion variables were simultaneously recorded at baseline. Perfusion variables included; PI, blood lactate level, central venous oxygen saturation (ScV02), and the difference between central venous carbon dioxide (PcvC02) and arterial carbon dioxide (PaC02) pressures (Pv-a C02). Arterial blood lactate will be measured at T0. The requirement of vasopressor was evaluated as yes/no.

## Results

A total of 36 patients fulfilling inclusion criteria were enrolled in our study, of whom 15 died and 21 survived. Twenty-one of the 36 patients required vasopressor and 15 did not. The cutoff of the PI value was ≤0.3 for predicting vasopressor requirement, resulting in a sensitivity of 100% and a specificity of 93%; AUC was 0.96 (95% CI 0.8-0.99), p < 0.0001. The cutoff of the PI value was ≤ 0.21 for predicting ICU mortality, with sensitivity of 86% and a specificity of 90%, AUC was 0.94 (95% CI 0.8-0.99), p < 0.0001.

## Conclusions

PI less than or equal to 0.3 is predictive of vasopressor requirement during early resuscitation of patients with severe sepsis and septic shock.
